# Qualitative exploration of using hormonal contraception on mental health and wellbeing: Diverse experiences and a call for more support

**DOI:** 10.1186/s12905-026-04583-z

**Published:** 2026-06-19

**Authors:** C. Jones, T. A. McAdams, Z. Akintoye, H. M. S. Zavos

**Affiliations:** 1 Social, Genetic and Developmental Psychiatry Centre, Institute of Psychiatry, Psychology and Neuroscience, King’s College London, Camberwell, London, UK; 2 Department of Psychology, Institute of Psychiatry, Psychology and Neuroscience, King’s College London, Guy’s Campus, London, UK

**Keywords:** Hormonal contraception, Mental health, Lived experience

## Abstract

**Introduction:**

Quantitative research examining hormonal contraception and its relationship with health, particularly mental health and wellbeing, has been inconclusive with mixed evidence regarding the magnitude and direction of associations. Qualitative approaches that focus on women’s perspectives and lived experiences may offer valuable insights into the inconsistent results reported. In the present study we take a qualitative approach to exploring the lived experiences of mental health and wellbeing among young women when using hormonal contraception.

**Methodology:**

Data were collected as part of the Twins Early Development Study (TEDS) when participants were aged 26. A total of 5594 women completed a questionnaire assessing mental and physical health. At the end of a section of a series of closed questions about menstruation and hormonal contraception, 626 respondents answered an open-ended question in which they were invited to share their thoughts on their related experiences. Of these responses, 272 specifically addressed links between mental health, wellbeing, and hormonal contraception and were included in the final analysis. Responses were analysed using thematic analysis.

**Results:**

Our analysis generated five themes representing positive, neutral, uncertain, and negative experiences of health and wellbeing whilst using hormonal contraception. The importance of choice and autonomy was emphasised. Many respondents reported feeling a lack of adequate care and support from healthcare professionals.

**Conclusion:**

Women report a range of experiences whilst using hormonal contraception that have positive and negative consequences on health and wellbeing. Healthcare providers should offer clear, detailed information and tailored support to women regarding the range of options available for hormonal contraception, and possible outcomes.

## Introduction

Over 874 million women of reproductive age across the world report using what are termed ‘modern contraceptive methods’ including hormonal contraceptives [1]. Hormonal contraception comprises a range of methods designed to prevent pregnancy including oral contraception, contraceptive patch, coil, injection, and implant [2]. Different methods vary regarding the type and amount of hormone; for example, some solely contain progesterone or oestrogen, and some contain both. Whilst hormonal contraception is primarily used as a method of birth control, it is also used as treatment for a range of menstruation-related conditions including endometriosis and heavy menstrual bleeding [3]. Despite being widely used, there are several reported possible side effects of hormonal contraception, including, but not limited to, weight gain, acne, mood swings, and headaches [4]. A growing body of predominantly quantitative research [5–11] has examined the impact of hormonal contraception on mental health and wellbeing. While standardized questionnaire measures have been useful in exploring factors such as, the impact of age when starting to use hormonal contraception or duration of use, they often lack the depth and nuance needed to fully understand individual lived experiences. The present study aimed to address this by adopting a qualitative approach.

There is inconsistent evidence regarding the impact of hormonal contraception on mental health outcomes. Some studies report an alleviation of symptoms of depression among certain groups of contraceptive users, including those with previous experience of mental health difficulties and those with menstrual health conditions [5]. A systematic review of randomized control trials and cohort studies found that among women who had previously experienced mental health conditions, fewer symptoms of depression were reported when using hormonal contraception [5]. However, the findings differed depending on prior mental health history: in those who previously did not have a diagnosed mental health condition, a slight increase in depression symptoms were reported. Research exploring menstrual health conditions too have reported on the positive effects of using hormonal contraception. A systematic review by Forslund et al. [6] of four randomized controlled trials found that combined oral contraceptives benefit women with Polycystic ovary syndrome (PCOS) by regulating menstrual cycles and improving quality of life. Some research evidence indicates that combined hormonal contraceptives can help treat the symptoms of endometriosis, however a lack of high-quality data limits the evidence base and certainty of these findings [7].

In contrast, a range of research studies have found no association between hormonal contraception use and depression [8, 9]. For example, in one systematic review and meta-analysis focusing on randomized control trials, no association between symptoms of depression and use of hormonal contraception was found [8]. However, it’s important to note that people are unlikely to self-select to take part in a hormonal contraception trial if they have previously had a negative experience of hormonal contraception. This may lead to biases in these results and explain why controlled trials often find no effect of hormonal contraception on mental health outcomes when observational studies sometimes do. Other studies have reported a negative association between use and depression, in particular when hormonal contraception is used from an early age. A study using UK Biobank data found oral contraceptives were associated with increased risk of depression when contraceptive use began during adolescence, and this was most significant in the first two years after beginning to use the contraceptives [10]. Similarly, a Danish prospective cohort study [11] found that using hormonal contraceptives during adolescence (defined as 15–19 years in the study) led to an increased likelihood of being diagnosed with depression and greater likelihood of using antidepressants within the 14 year period during which the participants were followed up. Yet, the impact is variable and, at times, inconsistently reported; negative associations are sometimes found with certain forms of contraceptives, but not universally across all types. Research has suggested that the contraceptive implant, hormonal Intrauterine Device (IUD) and contraceptive patch are associated with an increased risk of depression [5, 12]. More research teasing apart the reasons for these variations in symptoms is needed [13].

These findings therefore present a complex picture, and the current literature is limited by several factors. People who take hormonal contraception are heterogeneous and quantitative studies conducted to date have not been able to capture the full picture. In addition, extant research focuses mostly on depression symptomatology; looking at mental health and wellbeing more broadly would add further insight into the range of experiences amongst people using hormonal contraception. This may have contributed to the apparent contrast between published evidence and anecdotal perspectives from individuals with lived experience, as well the limited qualitative data exploring lived experiences and perspectives of those using hormonal contraception. Martell et al.’s [14] survey of 188 people who had used hormonal contraception found that the most common reason for switching hormonal contraception was experiencing side effects. From open-text responses, mood changes were commonly cited, providing some lived experience insight. However, the sample comprised people who had used hormonal contraception at any point in their life, so recall bias and changes in the doses and types of hormonal contraception available may have influenced the results. A systematic review of 42 studies exploring reasons and arguments for not using hormonal contraception captured reasons beyond a possible direct impact on mental health, and included impact on libido, worries about reduced fertility, concerns about the wider impact of hormones, and feeling unsupported with concerns by healthcare providers [15]. This points to the wider possible impact of hormonal contraception on wellbeing, particularly due to the stress or worry of perceived side-effects. Yet, this research only provides insights into why hormonal contraception use is rejected, therefore not capturing the full spectrum of experiences. Qualitative research exploring perspectives of using hormonal contraception have found in an area with a high rate of young maternal age pregnancies, concerns were reported about ‘unnatural’ hormones in hormonal contraception, and tensions emerged between the ‘control’ afforded from using hormonal contraception versus a desire to experience ‘natural’ menstruation [16]. Mistrust – sometimes due to misconceptions – of hormonal contraception has also been reported to influence young people’s thoughts about using hormonal contraception in a small qualitative study [17]. However, these research studies did not explore the possible wider impact on mental health and wellbeing.

In the present study we seek to better understand the individual lived experiences of women using hormonal contraception. This is useful in firstly providing insight into effects beyond, for example, depression symptoms, which are commonly explored, and secondly, to inform future avenues of research on this topic shaped by lived experiences. In summary, our research aimed to explore how women in young adulthood describe their mental health and wellbeing whilst using hormonal contraception.

## Methods

### Participants

The TEDS participants were recruited from 1994 to 1996 from national birth records in England and Wales, with an original sample size of 16,000 twin-pairs [18]. For the purposes of the TEDS-26 mental health study, the retained 10,328 twin pairs and 306 unpaired twins were invited via email and post between July 2021 to December 2022. Participants age ranged between 24 and 29 years-old with an average age of 26 years-old at the time of data collection. The current research consisted of a sub-sample of individuals (11% of those eligible) who identified their gender as female (*N* = 5594) and who responded to an open text question (*N* = 626). Of these, data from 272 participants whose responses to the open question related to hormonal contraception, their mental and physical health, and wellbeing, were analysed.

Self-reported demographic characteristics of participants included in this study are presented in Table 1. The sample includes a range of relationship statuses and sexual orientations, though the majority of respondents identified as heterosexual and in a relationship. Most participants identified their ethnicity as White British, and there was limited ethnic diversity in the subsample. The proportion of respondents who hold a university degree – either undergraduate only or undergraduate and postgraduate – is comparable to national estimates [19]. However, higher education attainment of those who responded to the open-ended question was higher than the national average.

**Table 1:** Self-reported demographic data at age 26

	Total sample of eligible respondents (*N* = 5594)		Included in thematic analysis (N = 272)		X²	*p*
	*N*	Proportion (%)	*N*	Proportion (%)		
Relationship status						
Single	1805	32.3	71	26.1	8.44	*n.s.*
In a relationship	3374	60.3	174	64		
Married or civil partnership	372	6.6	25	9.2		
Other	18	0.3	0	0		
Missing	25	0.5	2	0.7		
Sexual orientation						
Heterosexual	4591	82.1	221	81.3	17.91	< 0.01
Homosexual	185	3.3	2	0.7		
Bisexual	435	7.8	29	10.7		
Pansexual	74	1.3	9	3.3		
Asexual	45	0.8	0	0		
Fluid	22	0.4	0	0		
Self-define	28	0.5	2	0.7		
Missing	214	3.8	9	3.3		
Ethnicity						
Asian Chinese	13	0.2	4	1.5	13.14	*n.s.*
Asian Indian	65	1.2	0	0		
Asian Pakistani	42	0.8	0	0		
Asian other	13	0.2	1	0.3		
Black African	24	0.4	1	0.3		
Black Caribbean	31	0.6	4	1.5		
Mixed white and Asian	57	1	0	0		
Mixed white and black Caribbean	62	1.1	0	0		
Mixed other	37	0.7	1	0.3		
White British	5147	92	258	94.9		
White Irish	25	0.4	0	0		
White other	53	0.9	0	0		
Any other	15	0.3	2	0.7		
Missing	10	0.2	1	0.3		
Highest level of qualification						
GCSEs	430	7.7	6	2.3	25.55	< 0.01
A levels	773	13.8	33	12.1		
Undergraduate degree or higher	3613	64.6	211	77.6		
Other	619	11.1	18	6.6		
Missing	159	2.8	4	1.2		

### Measures

The TEDS-26 online Mental Health Questionnaire was administered via Qualtrics, an online questionnaire platform. The questionnaire comprised of standardized measures of mental health and questions designed specifically for the study. More information is available from the TEDS data dictionary (https://datadictionary.teds.ac.uk/studies/measures/measures.htm).

Participants were asked demographic questions. Participants who responded ‘female’ to the demographics question ‘which gender do you identify with?’ were then administered questions on hormonal contraceptive use devised for the purpose of the wave 26 data collection. The hormonal contraception section of TEDS-26 included a combination of tick-box answers, closed questions and one open-ended question. The questions explored prior and current use of hormonal contraceptives, premenstrual symptoms, and participant interest in future research related to hormonal contraception and mental health. All questions can be viewed online (26 Year Study Measures). The final question of the section asked, “If you have anything you would like to add on this topic, please feel free to add some text in the space below”, and from this question, the present dataset for qualitative analysis was retrieved. Our analytical plan was pre-registered on Open Science Framework (OSF Registries | Exploring experiences of support seeking and mental health while using hormonal contraception in young adulthood). An edit was made to use thematic analysis instead of qualitative content analysis to account for the complexity of responses, enabling a more in-depth analysis of the participants’ perspectives.

### Premenstrual symptoms impact survey

The PMSIS *(PMSIS; Wallstein et al.* [20]*)* is a six-item scale assessing the extent to which premenstrual symptoms impact quality of life. It focuses on how these symptoms affect physical, social, and cognitive functioning, as well as mental health. The six items are rated on a 5-point likert scale from ‘None of the time’ to ‘All of the time’. A sample item is ‘During your last premenstrual period, how much of the time did your premenstrual symptoms limit your ability to concentrate on work or daily activities?’. The PMSIS has been validated and found to be effective in distinguishing between people with clinically relevant levels of premenstrual symptoms.

### Procedure

The responses pertaining to the research questions contained 94,770 characters (minimum 32 characters and maximum 2000) with an average of 150 characters. These responses were analysed using thematic analysis on NVivo, a qualitative analysis software.

### Ethical considerations

TEDS has ethical approval from Kings College London Research Ethics Committee (References: PNM/09/10–104 and HR/DP-20/21–22060). Informed consent was obtained before data collection at every wave.

### Thematic analysis

Thematic analysis is described as the process of interpreting, analysing and generating themes within datasets [21] and was chosen as the method of analysis as it provides a framework for exploring how individuals make sense of their experiences. Thematic analysis is beneficial due to the ability to account for similarities and differences within datasets and participant experiences [22].

An inductive, data-driven approach was adopted. Codes and themes were generated prior to forming overarching conclusions and answers to the research questions. Braun and Clarke’s [21] six phases of thematic analysis were followed, whereby firstly familiarisation took place by repeatedly reading the data, aiding the interpretative process. Using NVivo software, explicit codes and underlying patterns were identified through the formation of semantic and latent codes [23]. To ensure reflexivity and credibility of the thematic analysis process, before analysing the full dataset, the first 25% of responses were coded by all investigators, whereby reflections about the generated codes were shared and checked for over-interpretations. Analysis was then led by the first and third authors. Semantic and latent codes were then grouped around re-occurring patterns within the data to generate themes. Example codes included ‘not acknowledged by GP’, ‘pill negatively impacted mood’, ‘stopped pill due to impact on mental health’ and ‘wish for more support’. The themes were reviewed for internal consistency and independence through assessing the quality of the themes, for credibility, dependability, confirmability and transferability [24]. To assess for quality, discussions between the project researchers regarding whether the interpretations fit with participant experiences took place and the thematic map was shared to account for the generated themes, to further explore the link between the data and overall conclusions. Verbatim quotes have been selected to accompany the presented themes and subthemes, in line with qualitative quality control guidelines [25].

### Positionality statement

The two authors who carried out the thematic analysis acknowledge that their age, gender identity, and experiences of hormonal contraception enable them to simultaneously occupy both an insider and outsider identity in relation to the participants [26]. Continued detailed discussions during data analysis enabled an exploration of how our identities and perceptions shaped our interpretation of the data.

## Results

Participants’ current contraceptive use and self-reported premenstrual symptoms are presented in Table 2. Note that the open-text responses provided perspectives on hormonal contraception experiences at any point, not just amongst those currently using hormonal contraception. The combined contraceptive pill was the most popular hormonal method. The subsample were less likely to be using the contraceptive pill currently and more likely to be using condoms or a fertility app for contraception.

A one-sample t-test showed that participants who provided a text response to the open-ended question reported higher scores in terms of the impact of premenstrual symptoms on quality of life compared to the overall sample, *t*(268) = 4.18, *p*<.001. The subsample were also more likely to cite using hormonal contraception to control for physical or emotional symptoms, or to avoid side-effects of other forms of contraception.

**Table 2:** Hormonal Contraception use, reasons for using contraception, and Premenstrual Symptoms

	Total sample of possible respondents (*N* = 5594)		Included in thematic analysis (*N* = 272)		X²	*p*
	*N*	Proportion (%)	*N*	Proportion (%)		
Using combined pill	1357	24.3	48	17.6	44.92	< 0.001
Progesterone only pill	492	8.8	36	13.2		
Contraceptive injection	138	2.5	2	0.7		
Coil	498	8.9	38	14		
Hormonal coil	315	5.6	18	6.6		
Rhythm method	41	0.7	5	1.8		
Fertility app	309	5.5	30	11		
Condoms	1246	22.3	105	38.6		
Other contraception	416	7.4	14	5.1		
Major reason for using hormonal contraception:						
Avoid pregnancy	2874	51.4	175	64.3	25.76	< 0.01
Control physical symptoms	981	17.5	58	21.3		
Control emotional symptoms	383	6.8	28	10.3		
Avoid side effects of other forms of contraception	938	16.8	105	38.6		
					***t***	***p***
	***M***	***SD***	***M***	***SD***		
PMSIS total score	6.53	5.16	7.96	5.62	4.18	< 0.001

**Fig. 1:**
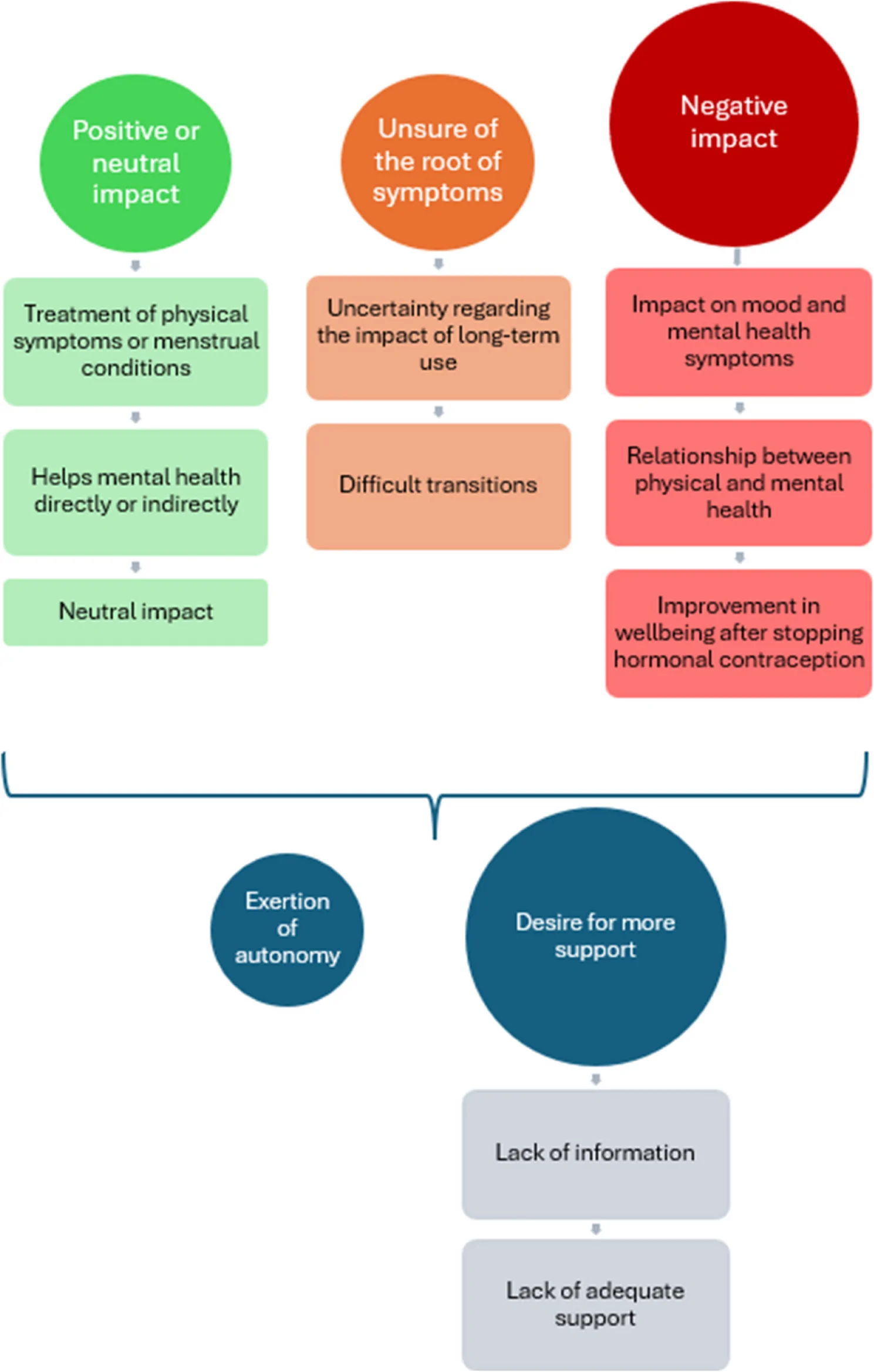
Thematic map showing the five themes and ten subthemes which describe participants’ experiences of their health, wellbeing, and support seeking in relation to hormonal contraception use

Five themes explain the participants’ responses to the open-ended question. The first theme ‘Positive or neutral impact of hormonal contraception on health and wellbeing’ explains participants’ accounts where using hormonal contraception was reported to benefit wellbeing or overall health, or no differences in mental health had been observed. The second theme ‘Unsure of the root of symptoms’ represented that for some participants, the effects of hormonal contraception were either unclear, or represented a mix of positive and negative experiences. The third theme ‘Negative impact of hormonal contraception on health and wellbeing’ relates to the negative effects participants reported experiencing when using hormonal contraception. More respondents mentioned negative effects than positive effects, as represented visually in the thematic map. These themes encompass the diversity and heterogeneity of responses across the sample. They also demonstrate the complexity of understanding the ways in which women experienced their mental health, and health more broadly, whilst using hormonal contraception. The fourth theme ‘Exertion of autonomy’ describes the importance participants placed on choice regarding using, or discontinuing to use, hormonal contraception. Finally, the fifth theme ‘Desire for more support’ explains the narrative which frequently emerged that support for mental health when using hormonal contraception was insufficient. The themes and subthemes can be seen in the thematic map (see Fig. 1).

### Positive impact of hormonal contraception on health and wellbeing

The first theme ‘positive or neutral impact of hormonal contraception on health and wellbeing’ illustrates the range of benefits some of the participants reported when using different forms of hormonal contraception, and also a lack of impact on mental health, which is positioned as positive. Three subthemes make up this theme; Treatment of physical symptoms or menstrual conditions, Helps mental health directly or indirectly, and Neutral impact.

### Treatment of physical symptoms or menstrual conditions

Some respondents described the positive impact hormonal contraception had on their life; physical symptoms or conditions were improved through using hormonal contraception. For example, negative symptoms specifically associated with menstruation were eased. Participants outlined “I love my IUD hormonal coil. It’s significantly reduced my PMS and cramps which I hated” and “I have really bad cramps which the pill stopped.” The alleviation of physical symptoms was reported to have a beneficial effect on wider quality of life; “The pill makes my period so much lighter and allows me to plan my life easier.” In addition to other reported health benefits, the pill is sometimes recommended as a treatment to address skin problems. Several participants shared positive experiences of this use, noting that the “mini pill helps with my acne”.

A subgroup of participants used hormonal contraception to address conditions, reporting how the contraceptives helped alleviate their symptoms, for example “Hormonal contraceptives used for help with PCOS symptoms”. Likewise, another participant reported that the “Hormonal coil is used to reduce symptoms of endometriosis”.

### Helps mental health directly or indirectly

For some women, they detailed the perceived role of hormonal contraception in mood regulation and overall mental health. One respondent outlined “I like the pill because I always know what kind of mood I will be in on each day, it is like clockwork for me”, representing the benefits of the consistency felt whilst using hormonal contraception. Another participant commented that “the hormonal coil which I had fitted three years ago seemed to to reduce mood swings and improve my emotional regulation during my periods and in general.” For some, the positive impact on their mental wellbeing was particularly notable: “I have had no emotional/mental side effects from either combined or POP pill, implant or ring. This is fortunate as without any contraception, I am incredibly emotional, mood swings, depressed, suicidal and with very heavy bleeding and pain.”

### Neutral impact

For some, there was an absence of specific positive or negative effects of using hormonal contraception; no differences were observed, and their mental health was reported to be consistent with their experiences without hormonal contraception. Participants reported “I don’t believe my contraception has any affect on my mental health, my mood has remained consistent regardless of whether I am on the pill or not” and “I personally dont feel that there is a link with my mental health and contraceptive use”.

Overall, this theme captures the perceived benefits of using hormonal contraception – not only on physical health or wellbeing, but on mental health – and also a continuation of prior health, with no effect observed.

### Unsure of the root of symptoms

Whilst some women expressed that hormonal contraception was largely or overwhelmingly a positive experience for them, others reported a mix of pros and cons of their use, or that they had observed changes over time, but could not pinpoint the cause – hormonal contraception, or otherwise. This theme captures the uncertainty expressed in some of the responses, and has two subthemes: Uncertainty regarding the impact of long-term use, and Difficult transitions.

### Uncertainty regarding the impact of long-term use

Participants discussed the implications of using hormonal contraception for an extended period of time, including the uncertainty they felt in disentangling what the impact of using it may be, compared to other causes. For some participants, this was expressed in terms of curiosity: “I’ve been on contraception for so long sometimes I think what would my mood be like not using it.” For others, this was the source of some concern; one participant mentioned that “I have been worried recently about the use of contraceptive pill and whether it will have long term effects for my body because I have been on it for so long.” Worries about long-term use were sometimes amplified by views on hormonal contraception shared online “there has been a lot in the media about how your whole personality can change when you stop taking it this also worries me a lot.”

Several women suggested that it was hard for them to pinpoint the effects of hormonal contraception, given that there are external factors at play too, and teasing apart the effects of these presents difficulties. One woman said “I had wondered if my hormonal contraceptives play a part in my mood, but it is hard to tell whether it could just be circumstantial.”

### Difficult transitions

Some participants discussed the impact of transitioning on and off hormonal contraception, rather than experiences whilst using hormonal contraception. Their responses highlighted the impact these periods of change and adjustment can have; “I had a really bad experience withdrawing from my pill. There is not enough known about this.” Worries were also expressed among those who disclosed they were trying to conceive. One participant suggested “I think it’s also important to talk about [the] toll on mental health when coming off contraception to conceive, and how the stress of trying to get pregnant affects women.” In addition, worries about the possible impact of hormonal contraception on fertility was seen as an additional mental burden “I have recently come off the pill in hope to try for a baby next year, and think in my head I’m now worried about the implications of being on the pill for so long will affect how I can conceive. . I think this has added to the stress of being ready to try.”

These responses capture the concerns expressed by some participants that they could not feel confident in whether using hormonal contraception had led to changes over time, and that for some participants, consistent messaging in the media reporting negative consequences of using hormonal contraception had cast doubt on their experiences. This theme also highlights that it is challenging to disentangle whether difficult experiences transitioning off of hormonal contraception is due to, for example, hormonal changes, or whether the life events and circumstances leading to a change in contraception have led to some of the negative experiences reported.

### Negative impact of hormonal contraception on health and wellbeing

In direct contrast to the first theme, the theme ‘negative impact of hormonal contraception on health and wellbeing’ encompasses the range of challenges the respondents described. More participants expressed experiencing a negative effect than positive impacts, or uncertainty. The theme has three subthemes; Impact on mood and mental health symptoms, Relationship between physical and mental health, and Improvement in wellbeing after stopping hormonal contraception.

### Impact on mood and mental health symptoms

Participants described the negative impact of hormonal contraception on symptoms of mental health in both specific and general terms. Some participants described a worsening of their mental health, in particular, citing depression or anxiety, such as explaining “I got incredibly depressed when on the pill.” Likewise, another participant reported experiencing “issues with my mental health - mainly anxiety and sometimes depression.”

Participants also referred to the impact on their mental health in general, rather than highlighting specific symptoms or conditions. For example, reporting that the pill had “a huge impact on my mood/ mental health” and “My mental health was negatively impacted by using the contraceptive pill”. For some participants, the impact they felt hormonal contraception had on their mental health was severe; one participant expressed “The pill for me took over my brain”. Other participants described different, but similarly severe, experiences, such as reporting “The pill literally ruined my mental health.”

For some women, the main difficulties they reported centred on changes in their mood, or symptoms such as irritability. One participant explained “I have wondered if my contraception contributes to low energy and sometimes low mood”. Some of the accounts reported serious changes to moods and behaviours, such as “my behaviour was more erratic and had more mood swings.” Another respondent reported “When taking the combined pill…my mood changed dramatically, I felt incredibly low and sad, irritable and angry…”. This was echoed in other accounts; one participant said on the pill they experienced “very low mood and dark thoughts when using it in the past.” Descriptions pertaining to mood rather than specific diagnoses are important to note as these may go undetected when looking for clinically relevant levels of symptoms, cut-offs, or diagnosed mental health disorders.

Some participants offered reasons for why they perceived hormonal contraception had influenced their mental health, for example, by outlining how they felt the timing of starting hormonal contraception played a key role “I am a firm believer that being prescribed contraceptive medication at a young age has effected my mental health as a teen and adult.”

### Relationship between physical and mental health

Some participants observed that the impact on their mental health was indirect and was due to experiencing side effects when using hormonal contraception. Examples of side effects included observing changes to weight, which had a subsequent impact on wellbeing: “During the time of being on the injection it made me gain weight which lead to lower self-esteem and image so I think inadvertently affected mental health.” Another participant described “Hormonal contraception made me gain weight, lose confidence, feel like a shell of myself.” Similar experiences were expressed by others; “I definitely noticed a correlation between the implant and my mental health/weight gain.” Others felt that hormonal contraception had a knock-on effect on their mental health due to impacting their libido; “The combined pill…caused a complete loss of interest in sex that negatively affected my relationships and mental health”. This highlights the importance of examining a broader range of perceived effects of hormonal contraception beyond direct effects on mental health conditions.

### Improvement in wellbeing after stopping hormonal contraception

Some participants reported that after stopping using hormonal contraception, they observed a positive change in mental health symptoms. For example, one participant described “After coming off the pill I noticed a dramatic improvement in my mood, my ability to regulate food intake”. Similarly, another participant reported “I’ve recently come off the combined pill and my mental health has improved a lot.” For some participants, a range of symptoms were reported to change: “I have recently come off of contraception and my anxiety has been reduced because of this change. I also feel like my moods are less intense and I don’t get as many cramps.”

This theme captures the range and severity of negative experiences reported by a number of participants. The participants often directly attributed their form of contraception – such as the pill – as the perceived cause of worsened mental health, though some participants described more serious or lasting effects than others.

### Exertion of autonomy

A narrative of exerting autonomy emerged within the participants’ responses. For some women, in the absence of adequate support, and due to reporting side effects when using hormonal contraception, they described making changes to their use of hormonal contraception. In doing so, they emphasised the role of their autonomous decision-making in their choice. One participant explained “I came of contraception because I noticed it had a negative effect on my mental health.” Other accounts echoed this too, for example “I recently came off my contraceptive pill because of it causing extreme low moods, anxiety and acne”.

Sometimes hormonal contraception was positioned by the respondents as unnatural, such as explaining “I didn’t like that it was regulating my natural cycle.”, and that without using it, they felt more attuned to their body. One participant reported “The progesterone only pill affected my libido (low) and I wanted to come off it to connect with body properly.” Another participant commented that they “enjoy the natural feedback you get from your body through the menstrual cycle and had missed that whilst taking the pill.” This fed into the wider narrative of a desire for autonomy and being in control of the choices they were making around menstruation.

However, an emphasis on exerting choice and autonomy also emerged in participants’ responses regarding deciding to use, or continue to use, hormonal contraception. One respondent said, “The benefits [of hormonal contraception] are undoubtedly the sexual freedom”, and another emphasised “it has been the best thing in the world to not have to worry about pregnancy”.

These different accounts encompassed in the theme of ‘Exertion of autonomy’ emphasise the role of their independent decision-making in participants’ use of hormonal contraception, and highlight tensions and contradictions within the dataset – that for some, it was freeing to choose to use hormonal contraception, and that for others, stopping use led to greater perceived freedom or feeling attuned with one’s body.

#### Desire for more support

A common response was that participants felt unsupported when it came to receiving help for their experiences of mental wellbeing and health whilst using hormonal contraception. As a result, a desire for more support was strongly expressed. This theme is comprised of two subthemes: Lack of information and Lack of adequate support by healthcare providers.

### Lack of information

Many of the responses outlined that there was not sufficient information accessible to guide respondents in their choices about hormonal contraception, or to help explain their symptoms. This was reflected in numerous accounts. One participant said “In the past, my mental health was affected by hormonal contraception but I had to make the connection myself. There was limited information out there, not even from my doctor or sexual health worker, I’m glad I put two and two together and did my own research.” Another participant commented “I do not think there is enough research on women’s health. Especially with hormones, mental health and how hormonal contraception affects mental and physical wellbeing.” This highlights the burden felt by people who menstruate to find the relevant information for themselves.

#### Lack of adequate support by healthcare providers

Another source of dissatisfaction was the quality of care provided by healthcare services. Many participants reported struggling to access support and then being disappointed by the outcome when they did have access to support. One participant described her struggles when trying to receive help for endometriosis: “Some of the major stresses around getting contraception is having access to it and having access to experienced doctors that understand the impacts of different contraceptive methods on endometriosis. Clinicians very dismissive.” Others too commented on the extent to which a lack of support had negatively impacted them, “I don’t feel there is enough help or guidance for girls and women, this is probably the most prominent aspect of my life that has mostly weighed on my mental health.” A few responses indicated that in the absence of adequate alternative options, they often found they were recommended a different hormonal contraceptive, after experiencing difficulties with one type: “The contraceptive pill deeply affected my mental health in a very negative way. No matter how many times I spoke to the doctor about it they would just recommend a different pill which did not help.” Participants emphasised the burden on women as a result; “I find it really depressing that women are expected to deal with these issues from such a young age with so little support.” Other accounts mirrored this frustration “I feel strongly it’s an area where women deserve to be listened to and have all options made clear to them. Too often girls are started on hormonal contraception in their teens and then stuck on them for many years of their lives.”

On the whole, this theme cohered together numerous responses that support was lacking and more needed to be done to better support young women making decisions around hormonal contraception in relation to their experiences of mental health and wellbeing.

## Discussion

Despite existing research on the impact of hormonal contraception on health, including mental health, little research has explored lived experiences, and impact on day-to-day life. We set out to address this in the present study, whereby we used data from a population-based twin study to explore experiences of health and wellbeing whilst using hormonal contraception. The results show diversity in experiences, which spanned from very negative to very positive. Participants’ experiences of using hormonal contraception also encompassed a desire for women to feel in control of their choices and bodily autonomy, in addition to a need for greater support to enable this. This research is particularly timely, given the recent rise in negative attitudes towards hormonal contraception and evidence suggesting that hormonal contraception use is declining [27]. The findings have important implications, yet it is also pertinent to acknowledge that more research is needed exploring diverse samples and paying particular attention to the experiences of marginalised groups.

Our emphasis on lived experience and the exploratory nature of the research provides insight into two key areas: what women highlight as important to share about their experiences, and what is desired in terms of experiences around choice and support. Regarding the former, the diversity in responses is important to emphasise; reported experiences ranged from very positive, to very negative. In terms of the more severe experiences reported, it is evident that a proportion of women experience a high degree of distress whilst using hormonal contraception that may not be adequately captured using standardized scales, or in studies whereby those who have negative experiences are unlikely to take part, such as randomized control trials. The present study’s use of qualitative methods enabled these experiences to be expressed. For those who reported mildly negative impacts, these too may go unnoticed in research. Our findings reveal that changes to mood were commonly experienced and highlights the need for future research to explore mental health at the symptom level rather than focussing, for example, on clinically relevant levels of depression. That hormonal contraception can have an adverse effect on emotional regulation has also been found in previous research [12, 28]. Qualitative research can complement large-scale quantitative studies by enabling a more in-depth understanding of the nuances of reported experiences.

Across the three themes of positive and negative effects on mental health, and unsure of the root of symptoms, there was a clear link between physical and mental health for many of the respondents. Experiences of period pain, menstrual-related conditions, heavy bleeding, changes to weight, and sex drive, all influencing mental health, support a holistic approach to understanding mental health whereby it is studied alongside physical health. It is clear from the present findings that the impact of hormonal contraception on mental health and health more broadly is heterogenous. It is therefore important to understand what is causing these differences. It could be that a predisposition toward mental health difficulties plays a role. In addition, with more information about what may be causing these differential effects, the diversity of experiences reported points towards the future potential of precision medicine, which considers genetics, environment, and lifestyle to tailor treatments [29], to predict which form of hormonal contraception might be best suited to each individual.

It is hard to establish if life events that occur at the same time as starting hormonal contraception, particularly during adolescence and young adulthood, are causing the reported mental health difficulties, or hormonal contraception. The present qualitative findings do not provide an understanding of possible causal links between hormonal contraceptive use and mental health nor of pathways between these two factors. Interestingly, a portion of respondents reflected on the long-term nature of using contraceptives, and that whilst they did not report any ill effects, they voiced concerns about whether they might feel differently if not using contraceptives. Often, initiation of hormonal contraception use coincides with a life period defined by multiple transitions. It is highly likely that a combination of factors is important, and longitudinal research will be important in elucidating these effects. However, that young people in the present sample wish to highlight the perceived impact of hormonal contraception on their mental health indicates that research needs to further our understanding of the potential causal role of hormonal contraception in mental health symptoms, particularly during adolescence and young adulthood.

Our findings add to the current literature by highlighting the importance of choice and autonomy for women using hormonal contraception. A dichotomy emerged whereby for some participants, the choice they were making was positioned to be empowering. For others, frustration about the burden being placed on them to be adequately informed was expressed. To enable women to exert their autonomous choice, more information needs to be readily available, such that young women have appropriate guidance about the implications of their decisions on hormonal contraceptive use. The importance of choice can be understood in relation to theories on locus of control; feeling in control of one’s life is associated with an increased sense of wellbeing [30]. Access to more information about the range of contraceptives may help develop a sense of control over one’s contraceptive choices and subsequently benefit mental health.

For some young women, their observed side effects of hormonal contraception such as a lower sex drive and weight gain had a reported impact on confidence and self-esteem. Self-esteem is often closely related to mental wellbeing due to the negative impact on confidence and self-worth, which can result in knock-on effects on interpersonal relationships and ability to navigate stressful periods [31, 32]. Similarly, prior qualitative studies have also found that weight gain was perceived negatively by women using hormonal contraception [33]. In terms of a lower sex drive, previous research presents inconsistent findings, whereby lower levels of sexual desire have been identified in some studies, e.g. [34], but not consistently in other research [35], indicating more research is needed to further explore the possible wider effects of hormonal contraceptive use, and what could be done to mitigate against any side-effects.

In line with our findings, previous literature has identified how women felt dismissed by medical professionals when voicing concerns about hormonal contraception use [36] and reported disappointment in a lack of information regarding women’s health [37, 38]. Recent research conducted by the Department of Health and Social Care investigating women’s experiences with healthcare professionals reported 84% of respondents described feeling not listened to by healthcare professionals and voiced a desire for more services specialised for women’s health conditions [39]. Combined with the findings from this study, this raises cause for concern that medical professionals may lack a detailed understanding of women’s health, perhaps due to the limited amount of information and research in this topic area, which points towards a need for improvements to be made both in terms of the prioritisation of research on menstruation including hormonal contraception, and support offered to women that is empirically informed.

One of our key findings that women feel unsupported when seeking help for challenges related to hormonal contraception indicates that they may be turning to support outside of the NHS, as found by Martell et al. [15], whereby women felt deterred from seeking professional advice. Prior research too has found that young people experience stigma around menstruation and struggle to reach out for support due to feeling that difficulties with periods are seen as normative and therefore not regarded as a health priority [40]. This is concerning as research on the role of social media in perceptions of hormonal contraception indicates that social media may contradict medical advice, can amplify negative attitudes towards hormonal contraception, and peer views play an important role in individual choices around contraception [41]. Previous research has emphasised the need for healthcare professionals to understand what information is available on social media about hormonal contraception to better support their patients [42]. Our finding that some women perceived the pill as being ‘unnatural’ and therefore stopped taking hormonal contraception reflects messages being voiced on social media [43, 44]. This insight is particularly important for healthcare professionals who play a key role in guiding young adults on their contraceptive choices, especially given the effectiveness of hormonal contraception for pregnancy prevention. This highlights the complex balancing of the possible negative effects of hormonal contraception for some women on aspects of mental health with the benefits, which, for many, include pregnancy prevention [14]. Future research should explore the effects of this on young people, for example, by exploring whether non-hormonal methods of contraception are used for pregnancy prevention after stopping hormonal contraceptive use, and whether this is seen to have an impact on mental health. Healthcare providers need to understand and react to these trends – which again leads to an increased emphasis on the need for clear guidance and information and ability for women to raise their concerns with healthcare professionals.

Our preliminary findings were presented at a Patient and Public Involvement and Engagement event, and the findings were used as prompts for group discussion. The themes of the discussion are available as a blog post [45]. Challenges of conditions such as PCOS, a lack of medical support, and a desire to be listened to and seen were raised. The event discussion mirrored our research findings and added further depth through detailed discussion.

### Strengths and limitations

In terms of the strengths of the present study, including women with conditions such as PCOS and endometriosis contrasts with much of the current body of literature, as often women with these conditions are excluded from samples [12, 13, 46]. In addition, by using a broad, open-ended question, this invited respondents to share what they specifically wished to highlight about their experiences, and as a result, a wide spectrum of experiences were reported. On the other hand, due to the general rather than specific nature of the question, it is possible that more respondents would have responded to a question specifically asking about their perceptions of their mental health whilst using hormonal contraception, so responses may have been lost due to the type of question posed. However, the present study has a large sample size for qualitative research, facilitating a broad spectrum of experiences to be included in the analysis.

The study has several limitations which are important to address. Using survey data restricted the opportunity to gain further information about the experiences described. Future research adopting in-depth interviews, or focus groups, would allow for follow-up questions to be asked for a fuller picture of participants’ experiences. In addition, interviews with healthcare professionals would be beneficial to identify the gaps in information regarding women’s health and explore how to improve support provided to women through healthcare services.

Another limitation of this study is that participants had an average age of 26 and 95% of the final sample identified as White. Therefore, the findings do not enable an exploration of how experiences of hormonal contraception might vary at different ages, and does not account for the experiences of those from different cultural backgrounds. Previous research has highlighted discrimination in healthcare settings; a survey of 2051 Black and mixed Black ethnicity participants found 65% reported experiences of discrimination [47]. Consequently, future research should strive for more inclusive samples. In addition, it will be useful for future research to specifically examine experiences of hormonal contraception and mental health during periods of hormonal change, such as after pregnancy, and perimenopause. It is also important to note that the respondents who provided an open-text answer had slightly raised PMS scores to the wider TEDS sample, indicating that those who feel that their premenstrual symptoms have a more significant impact on their life were more likely to respond.

## Conclusions

This project explored the impact of hormonal contraception on health and wellbeing among young women. A qualitative approach allowed for the collection of free-text responses and theme generation. Hormonal contraception had varied effects on health and wellbeing, with both positive and negative impacts reported. Many women felt under-supported by healthcare professionals when reporting the negative impact of hormonal contraception on their mental health. Future research with diverse ethnic groups and ages will enhance these findings. The findings contribute to a deeper understanding of the diverse experiences reported by women whilst using hormonal contraception and highlight the need for improvements in the healthcare system.

## Data Availability

Data for this study came from the Twins Early Development Study (TEDS). Researchers can apply for access to the data: [https://www.teds.ac.uk/researchers/teds-data-access-policy](https:/www.teds.ac.uk/researchers/teds-data-access-policy) Requests must be approved by the TEDS steering committee and have the support of a core TEDS collaborator.
